# Evolution of self‐incompatibility in the Brassicaceae: Lessons from a textbook example of natural selection

**DOI:** 10.1111/eva.12933

**Published:** 2020-03-03

**Authors:** Eléonore Durand, Maxime Chantreau, Audrey Le Veve, Roman Stetsenko, Manu Dubin, Mathieu Genete, Violaine Llaurens, Céline Poux, Camille Roux, Sylvain Billiard, Xavier Vekemans, Vincent Castric

**Affiliations:** ^1^ CNRS Univ. Lille UMR 8198 ‐ Evo‐Eco‐Paleo F-59000 Lille France; ^2^ Institut de Systématique, Evolution et Biodiversité (ISYEB) Muséum national d'Histoire naturelle CNRS, Sorbonne Université, EPHE, Université des Antilles CP 50 57 rue Cuvier, 75005 Paris France

**Keywords:** allelic diversification, balancing selection, dominance/recessivity interactions, plant mating systems, sexual reproduction

## Abstract

Self‐incompatibility (SI) is a self‐recognition genetic system enforcing outcrossing in hermaphroditic flowering plants and results in one of the arguably best understood forms of natural (balancing) selection maintaining genetic variation over long evolutionary times. A rich theoretical and empirical population genetics literature has considerably clarified how the distribution of SI phenotypes translates into fitness differences among individuals by a combination of inbreeding avoidance and rare‐allele advantage. At the same time, the molecular mechanisms by which self‐pollen is specifically recognized and rejected have been described in exquisite details in several model organisms, such that the genotype‐to‐phenotype map is also pretty well understood, notably in the Brassicaceae. Here, we review recent advances in these two fronts and illustrate how the joint availability of detailed characterization of genotype‐to‐phenotype and phenotype‐to‐fitness maps on a single genetic system (plant self‐incompatibility) provides the opportunity to understand the evolutionary process in a unique perspective, bringing novel insight on general questions about the emergence, maintenance, and diversification of a complex genetic system.

## 1 INTRODUCTION

The flower is the defining organ of angiosperms and is associated with the spectacular diversification of this group of plants (Soltis & Soltis, 2014). While flowers in extant species display an astonishing diversity of sizes, shapes, colors, and functions, a central feature of their ancestral state, hermaphroditism, has remained widespread (Sauquet et al., 2017), bringing pollen‐producing anthers in close physical proximity with the entry of female reproductive tracts (the stigma). It follows that the structure of this reproductive organ can cause self‐pollination to be very common, such that the potential for sexual reproduction by selfing is high. To avoid the deleterious effects of inbreeding depression (Charlesworth & Charlesworth, 1987), reproductive mechanisms preventing selfing have been repeatedly selected in the course of evolution (Barrett, 2010). Yet, the transition from outcrossing to selfing is one of the most commonly observed evolutionary transitions in flowering plants. In fact, a substantial part of the developmental and morphological diversity of flowers is believed to be driven by these reproductive strategies along the selfing‐outcrossing continuum (Barrett, 2010).

Self‐incompatibility (SI) is a widespread mechanism to avoid selfing in flowering plants, whereby fertile plants are capable of self‐pollen recognition and rejection and require compatible outcross pollen to produce seeds. At the phenotypic level, SI is typically determined by the segregation of a finite (often large) number of mating types, corresponding to as many distinct pollen–stigma recognition specificities. Beyond the sole avoidance of self‐fertilization, SI thus also prevents fertilization between unrelated individuals expressing identical recognition specificities (de Nettancourt, 1977). In populations where the number of such specificities is small, the seed set can thus be impaired, potentially causing viability issues in endangered species (Wagenius, Lonsdorf, & Neuhauser, 2007).

**Figure 1:**
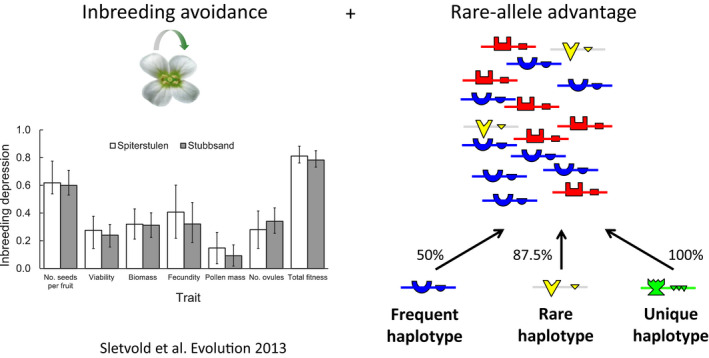
Self‐incompatibility is a textbook example of natural selection. Natural selection acts to remove nonfunctional variants in cases of strong inbreeding depression (as commonly observed in SI species such as *A. lyrata*, left, reproduced with permission from Sletvold, Mousset, Hagenblad, Hansson, & Agren, 2013). Even in populations devoid of SC variants, natural selection acts strongly by preventing fertilization between individuals expressing cognate specificities, hence causing negative frequency‐dependent selection on the genes controlling these specificities (right). The percentage of compatible mates is indicated for pollen expressing haplotypes with three contrasted frequencies, illustrating the rare‐allele advantage in this toy example

SI has been a model biological system in two distinct scientific communities. First in the plant molecular biology community, who developed SI as a model for how cells perceive external stimuli (self vs. outcross pollen) and mount a specific and appropriate cellular response (pollen tube rejection vs. acceptance). Identification of the SI genes in a handful of plant families revealed entirely different molecular and physiological processes, making it clear that SI arose independently several times (Franklin‐Tong, 2008). Much progress has been done in the last 15 years in some of these families toward a clearer understanding of how SI is controlled. In Brassicaceae, for example, SI is controlled by a highly diversified allelic series made of co‐adapted receptor–ligand combinations encoded by tightly linked genes in the so‐called “*S*‐locus” (generally *SCR/SP11* and *SRK* that encode for male and female specificities, respectively, Kusaba et al., 2001; Schopfer, Nasrallah, & Nasrallah, 1999; but see below for some interesting exceptions). The pollen determinant of SI is sporophytically controlled by the paternal parent and involves complex dominance interactions between SI alleles in heterozygotes (reviewed in Fujii & Takayama, 2018; Schoen & Busch, 2009). In Petunia, in contrast, SI is achieved by a very different mechanism involving complex non‐self‐recognition, where several linked paralogs gametophytically expressed in the pollen tube collectively determine the spectrum of recognition specificities (Fuji, Kubo, & Takayama, 2016). At the same time, SI has also been a model in the population genetics community, following the early realization by Wright (1939) that the mating success of individuals should be negatively correlated with the frequency of the SI specificities they express. This hypothesis of negative frequency‐dependent selection (Figure 1) has been the foundation for the rich theoretical literature on SI evolution, with the development of a series of models predicting the expected frequencies of SI alleles in natural populations, the conditions under which SI can be either maintained or lost, whether and how new SI alleles can arise given their peculiar genetic architecture, and how dominance interactions between SI alleles can evolve. The last 15 years have also been a period of rapid progress on this front, both to refine and considerably expand the theoretical predictions and to put them to the test by the genetic analysis of natural populations. In recent years, the field has been in the unique situation to merge these two scientific communities and take advantage of a genetic system in which the genotype‐to‐phenotype relationship has been considerably clarified, in a context where the phenotype‐to‐fitness map is also properly understood. Building upon these strong foundations, our group, along with others, has contributed to a better understanding of how SI phenotypes are controlled at the molecular level and a more precise understanding of how natural selection maintains the system in spite of the many forces that could lead to its disruption, eventually allowing its long‐term diversification in natural populations.

Here, we review this set of recent advances to illustrate how the study of such a model system can provide insight into more general biological phenomena such as the emergence of evolutionary novelty in a complex genetic system and the evolution of dominance. We highlight areas where more discoveries are likely to be made in the future. We focus in particular on how the scientific questions in the field have matured, spanning from the study of the signatures of natural selection to the emergence of functional and regulatory novelties.

## 2 THE RISE AND FALL OF SI SYSTEMS: THE SI GENES ARE MORE LABILE THAN WE THOUGHT

### 2.1 Conditions for the maintenance of SI are drastically restricted by a variety of different processes, leaving the existence of SI species an evolutionary puzzle

SI is one of many strategies (*e.g.,* herkogamy, dichogamy, dicliny, etc…) by which plants favor outcrossing and avoid selfing or crossing between related individuals (Barrett, 2002). Hence, self‐incompatibility is thought to have evolved in response to the negative selection generated by inbreeding depression, caused by the expression of recessive deleterious alleles in homozygotes (Charlesworth & Charlesworth, 1987). The conditions allowing the maintenance of SI are linked to the relative fitness of SI individuals compared with self‐compatible (SC) individuals. Inbreeding depression decreases the fitness of SC individuals because their selfed offspring are more prone to express recessive deleterious alleles, and this decrease is in proportion to the rate of self‐pollen deposited on stigmas. In some circumstances, this disadvantage can be offset because pollen expressing SC alleles enjoy universal compatibility: they do not trigger any SI response and can thus fertilize any potential mate in the population, while pollen expressing SI alleles will be rejected as a function of how frequent they are. Furthermore, SC alleles inherently benefit from the “automatic” transmission advantage of two‐thirds over SI alleles because they are transmitted in two copies by selfing (Fisher, 1941), and from their ability to fertilize all genotypes in the population (Charlesworth & Charlesworth, 1979). By investigating whether an SC allele can invade a population of SI individuals, Charlesworth and Charlesworth (1979) found that inbreeding depression has to be high (around two‐thirds when the number of SI alleles is large) in order for SI to be maintained, and the value of this threshold depends on whether the SC phenotype is due to loss of the pistil specificity, of the pollen specificity, or both. A limitation of this seminal model is that inbreeding depression was treated as a fixed parameter, and Porcher and Lande (2005) and Gervais, Abu Awad, Roze, Castric, and Billiard (2014) later showed, by including an explicit genetic architecture for inbreeding depression, that the presence of SC individuals in a population can lead to partial purging of deleterious mutations. This purging process further restrains the conditions allowing the maintenance of SI, especially when mutations have a strong effect on fitness. The distribution of fitness effects of mutations causing inbreeding depression thus appears as a key parameter to predict whether SI will be maintained or lost, and obtaining precise data on this poorly described distribution is a crucial priority for the field (Angeloni, Ouborg, & Leimu, 2011; Charlesworth & Willis, 2009). Beside these purely genetic processes, a series of studies have shown that the ecological conditions are also predicted to affect the maintenance of SI. Specifically, the invasion of SC mutants can be favored when the supply of compatible outcross pollen is limited (Jain, 1976; Porcher & Lande, 2005; Voillemot, Encinas‐Viso, & Pannell, 2019), which is a common phenomenon when partners are scarce (Baker, 1955; Burd, 1994; Pannell & Barrett, 1998; Willi, 2009) or when the genetic diversity at the *S*‐locus is low (Charlesworth & Charlesworth, 1979). It is worth noting that these factors are often correlated, in particular at the geographical margins of species distribution, where populations tend to be smaller, bottlenecked, and less dense, causing pollen limitation for SI individuals and favoring the spread of SC mutations (Busch, 2005; Griffin & Willi, 2014; Mable et al., 2017). Recently, Brom, Castric, and Billiard (2020) showed that because population subdivision leads to a reduction of the local diversity at the *S*‐locus (Schierup, Vekemans, & Charlesworth, 2000), it can also favor the invasion of SC individuals in SI populations, such that fragmented populations are more likely to lose SI than well‐mixed populations. Overall, theoretical models predict that loss of SI (evolution of SC populations/species) should occur repetitively within SI clades, in particular in conditions of decreased pollination efficiency or high habitat fragmentation (Brom et al., 2020; Busch & Schoen, 2008). Despite all the factors opposing its maintenance, SI remains widespread in angiosperms (Busch & Schoen, 2008; Ferrer & Good‐Avila, 2007; Igic, Bohs, & Kohn, 2006; Igic, Lande, & Kohn, 2008), leaving why such a system is so widespread and common an open question.

### 2.2 The breakdown of SI is typically recent and causal mutations can be identified

Selfing has classically been suggested to represent an evolutionary dead end under the argument that the lower level of standing genetic diversity of SC species may decrease their capacity to adapt in the face of changing environments (Stebbins, 1957), such that SC populations/species may be expected to remain short‐lived. At the macroevolutionary scale, SC species indeed show lower net diversification rates (Ferrer & Good, 2012; Goldberg et al., 2010), but distinguishing the relative contribution of differences in speciation versus extinction rates remains challenging with current phylogenetic methods. Both genetic and demographic factors probably influence the evolution and viability of SC populations, and there has been a rich theoretical literature on this topic (reviewed in Barrett, Arunkumar, & Wright, 2014). The shift to selfing has contrasted consequences on the efficacy of natural selection, which is expected to be weaker in SC populations due to a reduced effective population size and stronger selective interference effects due to linkage (Hartfield & Glémin, 2016), while at the same time strongly acting on deleterious recessive mutations because they are more exposed. Selfing is therefore expected to deeply modify the distribution of fitness effects of segregating advantageous and deleterious mutations, with major consequences on the genetic load and the adaptive potential (Arunkumar, Ness, Wright, & Barrett, 2015). A recent demo‐genetic model further showed that the expression of deleterious mutations in selfers can have a demographic cost that contributes to increase the rate of extinction (Abu Awad & Billiard, 2017).

As a result, most extant SC populations or species are believed to result from relatively recent shifts from SI with strict outcrossing to a SC status with a mixed‐mating or selfing regime. Indeed, a series of empirical studies using evolutionary analyses of the *S*‐locus have provided evidence for a relatively recent switch from SI to SC. These elements have been reviewed elsewhere (Vekemans, Poux, Goubet, & Castric, 2014), so we are only mentioning the sources of evidence here to illustrate their diversity. The evidences rely on: (a) the maintenance of ancestral polymorphism for nonfunctional *S*‐alleles at the *S*‐locus in SC species (in *Arabidopsis thaliana*, Bechsgaard, Castric, Charlesworth, Vekemans, & Schierup, 2006; Shimizu, Shimizu‐Inatsugi, Tsuchimatsu, & Purugganan, 2008; Tsuchimatsu et al., 2017; in *A. kamchatika*, Tsuchimatsu, Kaiser, Yew, Bachelier, & Shimizu, 2012; in selfing populations of *A. lyrata*, Mable et al., 2017); (b) the persistence of apparently intact sequences of *SRK* in SC species (*S_A_* haplotype in *A. thaliana*, Tsuchimatsu et al., 2010; in *Capsella rubella*, Guo et al., 2009; in *C. orientalis*, Bachmann et al., 2019; in *A. suecica*, Novikova et al., 2017; in *Leavenworthia alabamica*, Chantha, Herman, Platts, Vekemans, & Schoen, 2013); (c) the recent species origin by allopolyploidy associated with mating system switch (*A. suecica*, Novikova et al., 2017; *A. kamchatika*, Tsuchimatsu et al., 2012); and (d) the recent selective sweep detected at the *S*‐locus (in *L. alabamica*, Herman & Schoen, 2016).

There is a number of ways by which a SI allele can be inactivated. Yet, theoretical studies have also shown that not all SC mutations have identical probability to become fixed, and successful SC mutations should be associated with a set of defining functional features. In particular, loss of SI should evolve more frequently through nonfunctional mutations at the pollen rather than at the pistil component gene, because pollen SC mutations will benefit from increased mate compatibility as compared to pollen carrying functional SI specificities (Tsuchimatsu & Shimizu, 2013; Uyenoyama, Zhang, & Newbigin, 2001), which is not the case for pistil SC mutations unless there is strong mate limitation on seed set (Ehlers & Schierup, 2008). In addition, according to the phenomenon known as Haldane's sieve (Haldane, 1927; Turner, 1981), dominant SC mutations will be favored, which suggests that the loss of SI in sporophytic systems should evolve more readily when SC mutations occur in dominant *S*‐alleles. However, it is difficult to pinpoint the original causal mutation for the loss of SI in studied cases from the literature because SC species are typically exhibiting several nonfunctional mutations, and also because the loss of SI is expected to cause pseudogenization of genes required for SI recognition because of the relaxed selection, making it hard to distinguish causal from secondary mutations. Yet, a few empirical studies have identified putative original mutations in the pollen gene of dominant *S*‐haplotypes (in *C. orientalis*, Bachmann et al., 2019; in the *S_A_* haplotype of *A. thaliana*, with functional confirmation through engineered reverse mutation, Tsuchimatsu et al., 2010; in the *A. arenosa* parental genome of *A. suecica*, Novikova et al., 2017). Alternatively, interpopulation or interspecific pollination assays with functional SI donors of pollen or pistil were used to show that breakdown occurred mostly through the male function (Chantha et al., 2013; Tsuchimatsu et al., 2012), or both (Bachmann et al., 2019), and that SC haplotypes were dominant over SI haplotypes (Nasrallah, Liu, Sherman‐Broyles, Schmidt, & Nasrallah, 2007). Using genetic manipulations, the transfer of just the *S*‐locus genes from *A. lyrata* has enabled restoration of the SI system in several *A. thaliana* accessions, suggesting that in these accessions the downstream signaling cascade is complete and intact (Boggs, Nasrallah, & Nasrallah, 2009; Nasrallah, Liu, & Nasrallah, 2002; Nasrallah, Liu, Sherman‐Broyles, Boggs, & Nasrallah, 2004a). The same experiment led in other accessions did not result in restoration of SI, suggesting that additional modifiers of SI, putatively unlinked to the *S*‐locus, are segregating in *A. thaliana* (Nasrallah et al., 2004a). Additional evidence for unlinked modifiers comes from the study of North American *A. lyrata* in which some populations have recently shifted to selfing through independent losses of SI, possibly caused by modifier loci unrelated to the *S*‐locus itself (Mable et al., 2017). Recent interpopulation crosses suggested that at least two interacting unlinked loci underlie the loss of SI in these populations (Li, Kleunen, & Stift, 2019).

Once the breakdown of SI has occurred in a mutant individual, the strength of the genetic load may constrain the spread of the associated mutation. North American *A. lyrata* SI populations display lower inbreeding depression compared with European SI populations, which may have favored the transition to selfing once the breakdown mutation occurred (Carleial, Kleunen, & Stift, 2017). This result suggests a weaker limitation for selfing evolution in North American populations, which is consistent with the multiple selfing shifts observed in North America (Mable & Adam, 2007; Mable, Robertson, Dart, Berardo, & Witham, 2005). Using experimental populations of *Linaria Cavanillesii*, Voillemot et al. (2019) observed that SC tended to spread rapidly after SC individuals were introduced in a SI population with high inbreeding depression. SC individuals tended to have higher seed set and a higher outcross siring success compared to SI individuals, and the difference between them depended on pollinators availability.

### 2.3 Recruiting new SI genes may actually be straightforward

It is clear that SI can be lost in a variety of different ways. But how did it emerge in the first place? In Brassicaceae, the *SRK* gene, encoding for the female specificity in SI response, was proposed to have emerged from rearrangements of a large gene family that has specifically expanded in the Brassicaceae, providing the raw material for novel domain combinations (Zhang, Wang, Yuan, Tian, & Yang, 2011). The male determinant *SCR* has structural features reminiscent of antimicrobial peptides and may thus have been exapted from specific recognition functions toward defense against pathogens (reviewed in Kachroo, Nasrallah, & Nasrallah, 2002). *SCR*‐like and *SRK*‐like sequences do not seem to be randomly distributed in Brassicaceae genomes, but are often found in clusters containing both *SCR*‐like and *SRK*‐like sequences (Zhang et al., 2011). This specific co‐localization further suggests that *SCR* and *SRK* could have been recruited from pre‐existing signaling systems not related to self‐pollen recognition, possibly involved in defense against pathogens (Sanabria, Goring, Nürnberger, & Dubery, 2008; Zhang et al., 2011).

The process of de novo emergence of a novel SI system has been recently studied in the genus Leavenworthia (Brassicaceae), where SI is controlled by a non‐*SRK‐SCR*‐based *S*‐locus. In Leavenworthia, the self‐pollen recognition mechanism seems to have been acquired secondarily from *SRK* and *SCR* paralogues, named *Lal2* and *SCR‐like* (*SCRL*), respectively (Busch, Sharma, & Schoen, 2008; Chantha et al., 2013). *Lal2* and *SCRL* are phylogenetically divergent from *SRK* and *SCR*, suggesting that the duplication is ancient, possibly dating back to before *SRK* and *SCR* ancestors acquired their function in SI. The recruitment of *SRK‐SCR* and *Lal2*‐*SCRL* in SI signaling would thus represent independent acquisitions of the SI function (Chantha et al., 2013). The genomic region containing the ancestral *SRK‐SCR*‐based *S*‐locus has also been lost. This observation raises the question of which event occurred first, between the loss of the ancestral *SRK‐SCR*‐based *S*‐locus and the time of recruitment of *Lal2* and *SCRL* in SI function. This order has important phenotypic consequences because it will determine if Leavenworthia lineages went through a self‐compatible transition before the secondary acquisition of SI or if both functional *S*‐loci have coexisted. Intriguingly, Chantha et al. (2017) recently demonstrated that a rare functional *SRK‐SCR*‐based *S*‐haplotype was segregating at the same genomic location as *Lal2*‐*SCRL*, probably as a result of a translocation or recombination event. This tends to favor the scenario where the functional *SRK‐SCR*‐based *S*‐locus was not entirely lost during the recruitment of *Lal2* and *SCRL* for SI system, suggesting the transient coexistence of two *S*‐loci with different evolutionary origins.

Together, these results illustrate that the raw material for the emergence of novel *S*‐loci can arise through different evolutionary trajectories, possibly using functionally related gene families and a repertoire of “pre‐adapted” *S*‐receptor‐like genes in Brassicaceae. Similarly, independent origins of two distinct *S*‐RNase‐based gametophytic SI systems have been suggested in the Rosaceae family, generating the coexistence of distinct self‐recognition (genus Prunus) and non‐self‐recognition systems (genera Malus and Pyrus) within the family (Aguiar et al., 2015). In Papaveraceae, the SI reaction relies on a programmed cell death process, which is highly conserved across angiosperms, to the point that the Papaver SI genes can function in *A. thaliana* (de Graaf et al., 2012; Lin, Eaves, Sanchez‐Moran, Franklin, & Franklin‐Tong, 2015). It is tempting to speculate that the high level of conservation of this machinery may facilitate the recruitment of “ready‐to‐use” systems, enabling the repeated evolution of SI in different plant species. Overall, although the de novo emergence of SI systems appears to be rare, we believe that further research on additional SI clades has great potential to challenge our views about the emergence of novel complex phenotypes.

## 3 THE ORIGINS OF AN EXTRAORDINARY LEVEL OF DIVERSITY: A KEY ROLE FOR THE GENETIC ARCHITECTURE IN THE DIVERSIFICATION PROCESS

### 3.1 There are many alleles in natural populations

One of the most striking features of SI systems is the very large number of *S*‐alleles co‐occurring within populations (typically on the order of 10–35 alleles in intra‐population samples, Castric & Vekemans, 2004), and up to 50–100 alleles overall in some SI species (Lawrence, 2000). Together with the extreme sequence divergence among *S*‐alleles for the pollen and pistil functional components, leading to high degrees of trans‐specific and even trans‐generic polymorphisms (Castric & Vekemans, 2004; Vekemans & Slatkin, 1994; Wright, 1939), this makes the SI genes some of the most polymorphic loci in plant genomes. Because of such diversity, genotyping the *S*‐locus in natural populations of Brassicaceae has been a major methodological challenge. Additional uncertainty comes from the coexistence of homozygous and heterozygous *S*‐locus genotypes made possible by the dominance relationships among *S*‐alleles in sporophytic SI (Schierup, Vekemans, & Christiansen, 1997). Initial exploration of allelic diversity in natural populations involved cloning and sequencing approaches in association with controlled pollinations and segregation analyses (in *A. lyrata*, Mable, Schierup, & Charlesworth, 2003; Schierup, Mikkelsen, & Hein, 2001; in *A. halleri*, Castric, Bechsgaard, Schierup, & Vekemans, 2008; Castric & Vekemans, 2004). In order to perform systematic surveys in natural populations, approaches involving PCR amplification using sets of general degenerate primers designed in conserved regions of the genes were developed, followed by either direct sequencing using Sanger (Edh, Widén, & Ceplitis, 2009), separation of alleles using restriction enzyme profiling (Charlesworth, Awadalla, Mable, & Schierup, 2000; Schierup, Bechsgaard, Nielsen, & Christiansen, 2006), the single‐strand conformation polymorphism method (SSCP, Joly & Schoen, 2011) or more recently amplicon‐based NGS technologies (Jørgensen, Lagesen, Mable, & Brysting, 2012; Mable et al., 2017). In cases where reference sequences of *S*‐alleles were known, rapid population surveys could be performed using presence/absence PCR screening with *S*‐allele‐specific primers followed by gel electrophoresis (Bechsgaard, Bataillon, & Schierup, 2004; Llaurens et al., 2008). This methodological challenge has recently been overcome by the development of new bioinformatic approaches to infer *S*‐allele genotypes from raw reads of individual shotgun sequencing (Genete, Castric, & Vekemans, 2019; Mable et al., 2018) which allow obtaining accurate discrimination between homozygous and heterozygous *S*‐locus genotypes (based on read depth) and identification of new *S*‐allele sequences by de novo assembly (Genete et al., 2019). Using published *S*‐allele sequences as well as analyzing available data from individual shotgun sequencing of *A. halleri* and *A. lyrata* populations, our current estimates of the number of *S*‐alleles species‐wide are 53 and 58, respectively, with 43 transpecifically shared alleles between them (X. Vekemans and V. Castric, unpublished data). The very large proportion of shared alleles among closely related species can be explained by the strong negative frequency‐dependent selection expected to maintain the ancestral polymorphism as well as by the adaptive introgression of *S*‐alleles after speciation (Castric et al., 2008; Schierup & Vekemans, 2008).

### 3.2 Models of allelic diversification

Since the seminal work by Wright (1939), it is now well understood that the maintenance of such an extraordinary diversity at the *S*‐locus is due to the balance between opposing forces: negative frequency‐dependent selection (a kind of balancing selection that favors rare alleles, Figure 1), mutations introducing new alleles, and genetic drift that drives allele loss. However, Wright (1939) assumed that new SI alleles result from a single mutation changing concomitantly the female and male specificities. Yet, in all plant families examined to date, the female and male specificities are actually encoded by distinct tightly linked genes (Takayama & Isogai, 2005). The constraints on the diversification process introduced by this peculiar genetic architecture of the *S*‐locus have been explored by a series of models.

**Figure 2:**
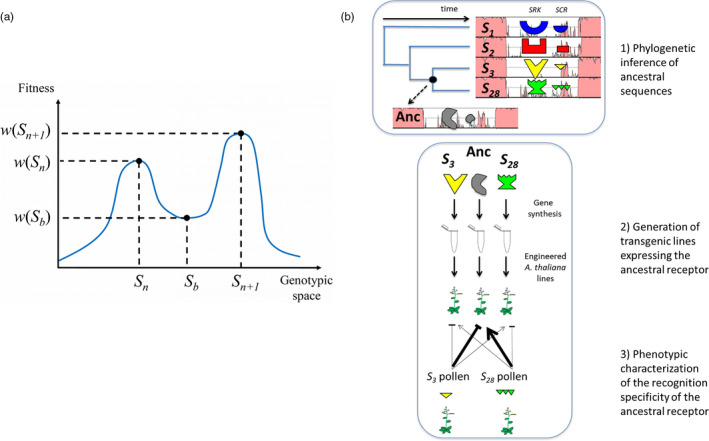
Theoretical (a) and empirical (b) approaches to study allelic diversification at the *S*‐locus. (a) Emergence of new *S*‐alleles in two‐component genetic systems involves the crossing of a fitness valley (see section 2.2). A simplified fitness landscape showing diversification in the two‐component genetic model of Uyenoyama et al. (2001) and Gervais et al. (2011). On the X‐axis is represented a simplified 1D genotypic space where the different haplotypes are placed. *S_n_*: ancestral SI haplotype, *S_b_*: intermediate SC mutant haplotype on the male component, *S_n+1_*: newly formed haplotype. Each genotype has an associated relative fitness (*w*) on the Y‐axis that depends on the combination of four evolutionary forces: transmission advantage of SC haplotypes, the universal compatibility of pollen *S_b_*, negative frequency‐dependent selection and inbreeding depression and proportion of selfed offspring. (b) An experimental approach based on ancestral protein resurrection to test evolutionary scenarios by which new SI alleles arise (see section 2.3 for details of the experiment). The asymmetrical recognition phenotype of the putative ancestor (Anc) shows that the *S_3_* pollen but not *S_28_* pollen has retained the capacity to trigger a SI reaction by the ancestral receptor. Hence, the ancestral recognition specificity has been conserved over a time at least equal to the divergence between *S_3_* and *S_28_*, rejecting models predicting rapid turnover of SI specificities. Note that these results also reject models of dual‐specificity intermediates, where the ancestral receptor would have recognized and rejected both *S_3_* and *S_28_* pollen

Uyenoyama et al. (2001) considered that new SI haplotypes are formed by a mutation on the male or female determinant, creating a SC (self‐compatible) haplotype with a new pollen or pistil specificity. This first mutation is then followed by a compensatory mutation on the female or male determinant, restoring a SI haplotype in which the pistil specificity matches that of the pollen. In this model, the emergence of a new *S*‐haplotype can be viewed as a special case of the crossing of a fitness valley since the intermediate SC haplotypes suffer from inbreeding depression while the newly formed double mutant (the new *S*‐haplotype) is initially unique in its population and therefore benefits from maximal mating success due to the advantage of the rare (Figure 2a). They showed that, in an infinite population, new SI haplotypes can emerge through mutations on the male determinant only, given inbreeding depression is very high and the rate of self‐pollination is moderate. They also showed that during this process the ancestral SI haplotype is almost always lost by competitive exclusion from its mutated SC haplotype, ultimately leading to the replacement of the ancestral allele by its derived version rather than an increase in the number of *S*‐haplotypes.

This model was extended by Gervais, Castric, Ressayre, and Billiard (2011), who further analyzed the model by Uyenoyama et al. (2001) and performed simulations in finite populations with recurrent mutations. They found that diversification can occur under a relatively wide range of parameters. However, they also showed that diversification is a self‐attenuating process, whereby the increase in the number of extant *S*‐alleles in the population makes the emergence of novel *S*‐alleles in turn less likely. Sakai (2016) refined this model by taking into account the evolution of the quantitative response of the rejection interaction, such that diversification proceeded through the transition from SC to SI by mutations enhancing or reducing the self‐recognition capacity of SI haplotypes. The rate at which new SI alleles were formed under this model decreased sharply when the SI alleles had evolved toward high self‐recognition capacity but was initially rapid, such that the resulting repertoire of SI alleles tended to be more important than in the standard models.

Other models have incorporated recent discoveries on the sharply distinct genetic architecture of the non‐self‐recognition system in *Solanaceae,* where the pollen specificity is encoded by a series of tightly linked paralogs collaboratively involved in specific inactivation of the toxic *S*‐RNAse proteins produced by pistils (Fujii et al., 2016; Kubo et al., 2010). Bod’ová et al. (2018) found that under this genetic architecture, diversification can proceed through SC as well as SI intermediate haplotypes, and that the level of “completeness” of the haplotypes (the number of other haplotypes compatible with a given haplotype) is a key factor predicting the probability that new *S*‐haplotypes arise. Harkness, Goldberg, and Brandvain (2019) further explored the role of gene conversion in generating new *S*‐haplotypes and considered that inbreeding depression is so important that SC intermediate haplotypes are absent from the population. They found that this gene conversion mechanism can drive a population to diversify more actively.

An important limitation of these models is that they consider single isolated populations only, while population subdivision is expected to affect strongly the overall segregation frequency and ultimate fate of SC mutants (Brom et al., 2020). Because SC mutants are key intermediate steps in models of allelic diversification at the *S*‐locus, we are currently expanding these models to explicitly consider the impact of population subdivision on *S*‐allele diversification (R. Stetsenko, V. Castric, & S. Billiard, in prep.).

### 3.3 Ancestral resurrection shows that SI recognition specificities are maintained over long evolutionary timescales and diversification occurs asymmetrically

The question of how new *S*‐alleles arise relates to the rate and evolutionary trajectories through which two‐component genetic systems like receptor‐ligand interactions can undergo functional diversification, currently a hot topic in evolutionary systems biology. A central issue raised by models of allelic diversification at the *S*‐locus is whether functional specificities either remain stable over evolutionary times, are subject to frequent turnover along allelic lineages (Chookajorn, Kachroo, Ripoll, Clark, & Nasrallah, 2004; Gervais et al., 2011), or entail promiscuous intermediates whose recognition specificity is initially broad but then progressively refines and specializes as diversification proceeds (Sakai, 2016). To distinguish among these possibilities, we recently used in Chantreau et al. (2019) a direct experimental approach that relied on probabilistic reconstruction of the last common ancestor of a pair of closely related yet functionally distinct *SRK* receptors in *A. halleri* (belonging to *S*‐haplotypes *S_3_* and *S_28_*; Castric et al., 2008). We engineered *A. thaliana* lines to express either the current (*S_3_* or *S_28_*) or ancestral versions of *SRK* alleles (Figure 2b), to characterize the rejection response of the ancestral receptor when it is pollinated with pollen expressing *S_3_* or *S_28_* specificities. The recognition phenotype of the ancestral receptor was strong and consistent toward *S_3_* but absent toward *S_28_* pollen, demonstrating that functional divergence of the receptor proceeded through an asymmetrical process (Figure 2b). Specifically, one of the two segregating alleles (*S_3_*) has retained the same recognition specificity as the ancestor, while the other one (*S_28_*) has functionally diverged and now represents a true evolutionary novelty. This observation shows that SI specificities can indeed be maintained over long evolutionary timescales and allow to reject models predicting rapid allelic turnover (possibly Chookajorn et al., 2004 and Gervais et al., 2011 under some circumstances) as well as models of promiscuous dual‐specificity intermediates (Matton et al., 1999).

## 4 NATURAL SELECTION AT THE *S*‐LOCUS

### 4.1 Experimental evidence for negative frequency‐dependent selection in natural populations

Once an *S*‐allele is introduced into a population, how does natural selection act on its evolutionary trajectory? There has been a long history of population genetics models addressing this question (*e.g.,* Ewens, 1964; Nagylaki, 1975; Wright, 1964, 1939), and the cloning of the SI genes in a number of species over the last 20 years has provided the unique opportunity to test the predictions from those models in natural populations. In the plant SI literature, a common approach to validate the negative frequency‐dependent selection hypothesis has been to test for isoplethy, that is, the expectation that all SI phenotypes should have equal frequency at equilibrium (Heuch, 1979; Lawrence, 2000). In finite subdivided populations, drift is expected to produce departures from the isoplethy expectation and simulation approaches have been used to take this into account when comparing observed distributions of allelic frequencies with theoretical expectations (Stoeckel, Castric, Mariette, & Vekemans, 2008; in the gametophytic SI system of Prunus). However, in sporophytic SI systems with dominance relationships among alleles, isoplethy will generate highly skewed allelic frequency distributions, with recessive alleles having larger population frequency than more dominant alleles. This makes explicit tests rather difficult to implement, especially since dominance interactions are often poorly documented (Billiard, Castric, & Vekemans, 2007; Schierup et al., 1997). Alternative and more powerful approaches consist of comparing observed and expected allele frequency changes across generations. Stoeckel, Klein, Oddou‐Muratorio, Musch, and Mariette (2012) implemented this approach in *Prunus avium*, a species with gametophytic SI, by testing for a negative correlation between allelic frequency change across generations and allelic frequency in the parental generation, as well as by testing for higher paternal success of individuals carrying *S*‐alleles with lower frequencies. Llaurens et al. (2008) implemented a comparable approach in a population of *A. halleri* (*i.e.,* with sporophytic SI), by computing the likelihood of alternative models taking into account drift only or drift with negative frequency‐dependent selection acting on the *S*‐locus, which confirmed the occurrence of selection. Focusing on the maternal success in experimental conditions of limited pollen availability, Leducq et al. (2010) showed that individuals with restricted availability of compatible pollen donors produced fewer seeds than individuals with higher proportions of compatible mates. Overall, it is clear that the population frequencies at which *S*‐alleles segregate in natural populations are strongly affected by the action of balancing selection.

### 4.2 Signature of balancing selection in SI genes

The long‐term evolution of SI genes also carries a variety of signatures of long‐term balancing selection. The first signature is an elevated level of sequence polymorphism, not only in terms of the number of allelic lineages maintained but also in terms of how divergent the lineages segregating within species typically are (Castric & Vekemans, 2004). The second signature is an elevated rate of evolution of the SI proteins. Rare mutants that just arose by mutations are expected to be favored if they modify the protein sequence and create novel SI specificities, while synonymous mutations should follow a neutral pattern. This should translate into an elevation of the ratio of the nonsynonymous (dN) to synonymous (dS) rates of evolution of the SI genes. Accordingly, several studies have revealed elevated dN/dS ratio for at least some codons of the SI genes in different species (in Arabidopsis and Brassica, Castric & Vekemans, 2007; in Leavenworthia, Herman, Busch, & Schoen, 2012; in Prunus, Vieira, Morales‐Hojas, Santos, & Vieira, 2007; in Solanaceae, Paape & Kohn, 2011). Interestingly, the location of these codons along the gene seems to match hypervariable regions, as well as the predicted atomic contacts between SCR and SRK in at least one allelic lineage of Brassica (Ma et al., 2016), suggesting that this pattern may help track the structural determinants of SI specificities. Also, the intensity of the dN/dS elevation for the most extreme codons seems to vary across species, possibly revealing differences in the intensity of allelic diversification. Indeed, the highest dN/dS were observed in Leavenworthia (Herman et al., 2012, dN/dS = 3.49) and Brassica (Castric & Vekemans, 2007, dN/dS = 2.49 vs. dN/dS = 1.49 in Arabidopsis), both of which are believed to have gone through relatively recent demographic bottlenecks that strongly reduced the number of allelic lines maintained at the SI genes. As shown in Gervais et al. (2011), a low number of *S*‐alleles is expected to cause a strong selection pressure for diversification. In these species, the bottleneck may thus have increased the recent dynamics of formation of new *S*‐alleles, resulting in a stronger signal of dN/dS. The third signature of long‐term balancing selection is the observation that many allelic lineages are shared across species or even genera, illustrating the strength of balancing selection to oppose their loss by drift (Takahata, 1990). This pattern of transpecific polymorphism is obvious in the Brassicaceae, where *A. halleri* and *A. lyrata* have a largely common set of *SRK* allelic lineages (Castric et al., 2008, see section 2.1), most of which are probably shared with the genus Capsella (Bachmann, Tedder, Laenen, Steige, & Slotte, 2018; Paetsch, Mayland‐Quellhorst, & Neuffer, 2006).

### 4.3 Local genomic effects

The negative frequency‐dependent selection acting at the *S*‐locus retains the segregating *S*‐haplotypes over long evolutionary times (Uyenoyama, 2003), leading to a local genomic excess of heterozygosity (Schierup et al., 2000; Takahata & Satta, 1998; Uyenoyama, 1997). Despite the strength of selection at the *S*‐locus, theoretical studies predict that its impact should be very localized on the linked surrounding regions (Schierup et al., 2001). This is explained by the extended length of its coalescent tree along which recombination events accumulate over time. Sequencing of natural populations in the genomic regions surrounding the *S*‐locus confirmed these theoretical expectations (Kamau, Charlesworth, & Charlesworth, 2007; Kamau & Charlesworth, 2005; Roux et al., 2013; Ruggiero, Jacquemin, Castric, & Vekemans, 2008). Such quantification has been carried out in the allogamous pair of sister species *A. lyrata* and *A. halleri* by sequencing fragments of the genomic region around the *S*‐locus. The overall levels of diversity directly measured at these regions were then corrected for demographic history (Roux et al., 2013, 2011), revealing that the excess of polymorphism explained by the linked selection concerns at most ~ 5kb around the *S*‐locus outside of the nonrecombining region. To date, such approaches relied on a discrete number of neighboring genes, preventing an accurate estimation of the size of the genomic region affected by linkage to the *S*‐locus, but long‐read sequencing technologies will render comprehensive exploration of this genomic region possible, in particular by including intergenic regions.

**Figure 3:**
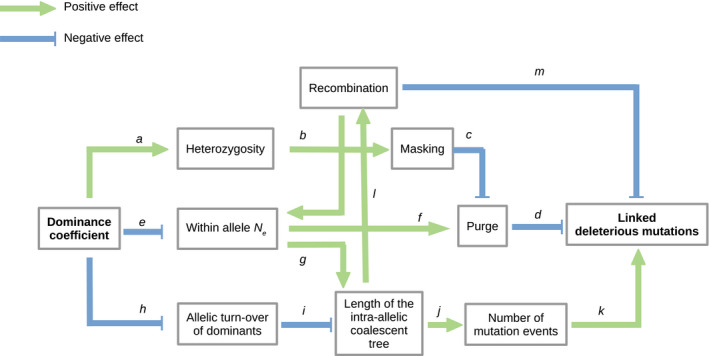
Theoretical predictions on the link between dominance and the load of deleterious mutations (see section 3.3). The formation of homozygous for the same *S*‐haplotype requires a cross between two heterozygotes sharing the same recessive and not expressed *S*‐allele. The formation of homozygous is thus impossible for the most dominant class. *b*. An increase in heterozygosity for an *S*‐haplotype also reduces the expression of recessive deleterious mutations associated with this haplotype. Yet, the recessive alleles occur more often at homozygous state so that their associated deleterious mutations are more often expressed. *c*. The purge of recessive deleterious alleles associated with *S*‐haplotype in the population is accelerated by their expression in the homozygous state. The latter is reduced when the dominance coefficient increases, leading to an increase in the genetic burden associated with dominant alleles; *d*. The stronger the purge is, the fewer deleterious mutations linked to an *S*‐haplotype are expected. *e*. In case of total co‐dominance between *S‐*alleles, the population is expected to be isoplethic for allele frequencies. With hierarchical dominance relationships, recessive *S*‐alleles can segregate at higher frequencies than the dominant ones, with a larger effective allele size than the dominant ones. *f*. For a slightly deleterious mutation linked to an *S*‐haplotype, the increase in the *N_e_.s* product (where *N_e_* is the effective population size and *s* corresponds to the effect of the mutation on individual fitness) will increase the efficiency of the purging of this mutation from the population. *g*. The total length of the intra‐*S*‐haplotype coalescing shaft increases for high *N_e_* values. An increased tree length will increase the population mutation rate theta (*j*), and the population recombination rate *rho* (*l*). *h*. Recessive *S*‐haplotypes are more likely to be lost by drift as compared to dominant ones that immediately benefit from negative frequency‐dependent selection when they occur at rare frequency within populations. *i*. Faster turnover of the recessive alleles reduces the average age of alleles compared to dominant alleles that segregate in the population over longer evolutionary times. *k*. A greater number of mutations in the coalescent tree are associated with an increase in the number of deleterious mutations. *m*. Recombination breaks the correlation in gene genealogies between the selected target (*S‐*locus) and the linked regions

The accumulation of deleterious mutations associated with loci under balancing selection has frequently been observed (see Llaurens, Whibley, & Joron, 2017 for a review). In the *S*‐locus region, the high level of heterozygosity coupled with a reduced recombination rate between *S*‐alleles is expected to favor the accumulation of deleterious mutations and transposable elements (Goubet et al., 2012). Enforced heterozygosity is expected to prevent their efficient purge, leading each *S‐*haplotype to accumulate with time a specific set of deleterious mutations. The dominance relationships between *S*‐haplotypes are also expected to have a strong impact on the accumulation of the associated genetic load (Llaurens, Gonthier, & Billiard, 2009b; Figure 3). Indeed, under some models of dominance, the rate of allelic turnover is predicted to differ between recessive and dominant haplotypes because the latter benefit from negative frequency‐dependent selection more readily when they become rare, leading recessive *S*‐haplotypes to be more likely to be lost by drift as compared to dominant ones (Billiard et al., 2007; Schierup et al., 1997). Thus, the dominant alleles, retained over longer evolutionary times, may accumulate more deleterious mutations than the recessive alleles. Moreover, recessive haplotypes are also more frequently observed at the homozygous state as compared to dominant ones (Schierup et al., 1997), providing more opportunities to purge the linked deleterious mutations for the recessive haplotypes than for the dominant ones. This dominance hierarchy among *S*‐haplotypes also influences haplotype frequencies within populations. Thus, recessive haplotypes reach higher frequencies than dominant ones (Billiard et al., 2007), further increasing the opportunities for purging to occur.

While the theoretical prediction is clear (dominant SI alleles should be associated with a more severe mutation load than recessive ones), empirical evidences of the sheltered load are scarce, notably because distinguishing the effect of inbreeding depression due to the mutational load spread throughout the genome from the effect of the genetic load specifically linked to the *S*‐locus is not straightforward (Glémin, Bataillon, Ronfort, Mignot, & Olivieri, 2001). This theoretical prediction was tested by (Llaurens, Gonthier, et al. 2009b) in *A. halleri*, who found that homozygotes for the highly dominant *S*‐haplotype *S_15_* had a lower fitness (as measured by a set of phenotypic traits) than homozygotes for the recessive *S*‐haplotype *S_1_* (where no load was detectable), consistent with the theoretical prediction. A genetic load associated with *S*‐haplotypes was also detected in *A. lyrata,* although the link with dominance was not confirmed (Stift et al., 2013). Overall, the existence of the sheltered load and its association with dominance remain controversial. The use of genomic data to pinpoint individual deleterious mutations (beside phenotypic quantification) holds great promise to resolve the genetic basis of this putative sheltered load. The fact that the *S*‐locus contains no protein‐coding genes in the Brassicaceae (beside *SCR* and *SRK* themselves) suggests that the deleterious mutations are more likely to be found in the immediate flanking genes rather than within the *S*‐locus itself.

## 5 MODELS AND MECHANISMS FOR THE CONTROL OF DOMINANCE

### 5.1 Evolution of dominance by modifiers is possible

As detailed above, the dominance relationships among SI alleles specific to sporophytic SI are expected to have a major impact on how the system evolves. But how do these dominance interactions themselves evolve, and how are they controlled? The evolution of dominance has been a recurrent topic in evolutionary biology (see Bagheri, 2006, for a review), and the study of SI in Brassicaceae has provided the first empirical evidence for a special kind of genetic element (“dominance modifiers”) that was proposed more than 90 years ago by R.A. Fisher (1928) in a different context but had remained elusive so far (Billiard & Castric, 2011). Indeed, it has been suggested that dominance could evolve more easily via dominance modifiers in populations where heterozygotes are frequent (Haldane, 1930), as for loci under balancing selection (Otto & Bourguet, 1999), such as the *S*‐locus. It is tempting to speculate that the evolution of the network of pairwise dominance interactions between *S*‐alleles should converge toward a linear hierarchy for two main reasons. First, theoretical studies showed that a mutation changing the dominance level without changing the allelic specificity (*i.e.*, acting as a dominance modifier) should be favored as long as it does not lead to co‐dominance (Llaurens, Billiard, Castric, & Vekemans, 2009a; Schoen & Busch, 2009). The dominance advantage is explained by the number of compatible mates, which is higher when one of the two alleles is masked in heterozygotes. Second, the accumulation of a sheltered genetic load may favor mutations that increase the dominance level, as recessive *S*‐alleles may form homozygous genotypes in which linked deleterious recessive mutations will be expressed (Llaurens, Billiard, et al., 2009a). Theoretical work also showed that dominance modifiers are more likely to fix if they arise in strong linkage disequilibrium with the *S*‐locus (Schoen & Busch, 2009), which gave hints to subsequent studies about where in the genome they were most likely to be found. Finally, because there is a difference in the strength of selection between the male and the female functions (whose magnitude depends on the extent of pollen limitation), the topology of the dominance hierarchies is expected to differ between pollen and pistils (Llaurens, Billiard, et al., 2009a; Schoen & Busch, 2009). Accordingly, in Brassicaceae, dominance among *S*‐alleles tends to be observed more frequently in pollen than in pistils (reviewed in Schoen & Busch, 2009).

### 5.2 The modifiers are small RNAs

Theory clearly predicts that dominance modifiers, if they exist, will be strongly selected for. But do they actually exist, and what is their molecular nature? A series of fascinating studies have revealed the molecular mechanisms driving pollen dominance interactions in Brassic, where *S*‐haplotypes are grouped into two dominance classes: the dominant class I and the recessive class II (noted Class I > Class II, Nasrallah & Nasrallah, 1993). Here, the dominance phenotype is explained by the transcriptional repression of recessive *SCR* alleles when combined with a more dominant allele (Kakizaki et al., 2003; Shiba et al., 2002). This transcriptional repression is caused by a 24 nt small RNA (sRNA, named *Smi* for *SP11* methylation inducer), produced by a hairpin RNA within the *S*‐locus, and acting in *trans‐* via de novo methylation of the promoter of the recessive *SCR* allele (Shiba et al., 2006; Tarutani et al., 2010). In other words, the dominant *S*‐haplotype produces a small RNA which specifically targets and represses the recessive *SCR* allele, and not the dominant allele, which is expressed normally.

**Figure 4:**
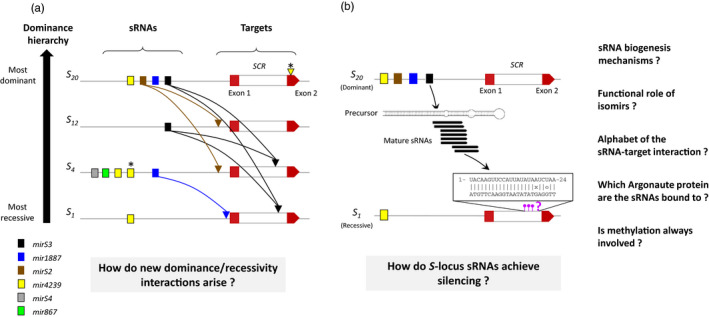
Dominance hierarchy among SI alleles in Arabidopsis. (a) Dominance can be explained by the evolution of a complex of sRNA regulatory network (see section 4.2). Schematic representation of the molecular regulatory network for four *A. halleri S*‐locus haplotypes chosen to be representative of the sRNA/target interactions identified in Durand et al. (2014), and ordered according to their dominance hierarchy. The repertoire of sRNA precursor families is represented by colored boxes, and *SCR* alleles are displayed for each haplotype. Target predictions, based on the sequence similarity between sRNAs and *SCR* alleles for all pairwise combinations, are displayed by curved arrows pointing to the target position (promoter or intron) when they are consistent with the phenotype, or marked by a star if not (here, only one predicted interaction opposed to the dominance phenotype was detected between *S_4_ mir4239* and exon2 in *SCR_2_*_0_). (b) Open questions on the mechanistic control of dominance (see section 4.4). Focus on one interaction to illustrate how dominance modifiers achieve silencing of the recessive alleles. The *mirS3* precursor of the dominant haplotype *S_20_* (represented by a black rectangle) is transcribed and produces a short hairpin structure, which is processed into mature sRNAs which recognize the intron of *SCR* of the recessive haplotype *S_1_* based on high sequence similarity between the mature sRNA and the target

This simple regulatory model was later shown to extend to more complex topologies of the dominance network, that is, when more than two levels of dominance exist. In *A. halleri* for instance, *S*‐alleles are grouped in at least four dominance classes with a linear dominance hierarchy generally observed within and between classes (Durand et al., 2014; Llaurens et al., 2008). Durand et al. (2014) showed that the molecular network controlling the dominance hierarchy among six *S*‐haplotypes was composed of at least eight distinct families of sRNA producing loci (for a total of 17 sRNA, one to five per *S*‐haplotype) and a diversity of targets located in different *SCR* regions (*e.g*., in the promoter or the intron, Figure 4a). Burghgraeve et al. (2018) confirmed that transcriptional silencing of recessive *SCR* alleles is also involved in *A. halleri* and clarified the base‐pair requirements for a given sRNA to target a given *SCR* sequence. In *B. rapa*, the linear dominance hierarchy among class II alleles (Hatakeyama, Watanabe, Takasaki, Ojima, & Hinata, 1998; Kakizaki et al., 2003; Yasuda et al., 2016) could also be explained by a sRNA‐based mechanism, but in this case with just a single sRNA precursor (*Smi2*) and its unique target. An important difference between the models by Durand et al. (2014) and Yasuda et al. (2016) is how new dominance/recessivity relationships arise. In Yasuda et al. (2016), the dominance network is assumed to have diversified via the joint accumulation of individual nucleotide changes at a single sRNA regulator or its one target site only, while Durand et al. (2014) proposed that de novo recruitment of new sRNA regulators and new target sites in the course of evolution is playing a key role, in addition to sequence modification of extant sRNA regulators and targets. It is possible that the relative importance of the two processes depends on the number of SI alleles among which selection is acting, such that a fixed set of sRNA regulators may suffice when few *S*‐alleles are involved (e.g., the class II alleles in *B. rapa*), while additional sRNA regulators may be necessary to allow the regulatory system to deal with the increased complexity of highly multiallelic systems (like in *A. halleri*).

Given this genetic architecture of pairwise gene regulatory interactions, how then does the dominance hierarchy evolve? Durand et al. (2014) showed that the most dominant *S*‐alleles reached the top of the hierarchy by evolution of the sRNA precursors they carry toward more generalism (enabling them to interact with more *SCR* targets), rather than by increasing their total numbers. On the other hand, the most recessive alleles tend to have more targets that are on average also more generalist because they tend to be targeted by multiple dominant alleles. In addition, cases of apparent redundancy have also been observed, with some dominant haplotypes targeting twice the *SCR* allele of the same recessive haplotype (e.g., *S_20_* is predicted to target *SCR_4_* at two different positions, *via* sRNAs produced by either the *mirS2* or the *mirS3* precursor it carries, Figure 4a). This observation raises the question about a potential additive effect, where both sRNAs could be needed to maintain the dominance phenotype. Alternatively, this redundancy could participate in the increase of the long‐term robustness of the network against loss of individual interactions. Overall, understanding the evolution of this fascinating regulatory network will ultimately involve understanding fitness consequences of the addition or removal of individual regulatory interactions between the sRNAs and their target sites.

### 5.3 Dominance and the breakdown of SI

An unexpected consequence of the machinery controlling dominance is that it has the potential to alter the conditions under which SI can either break down or be maintained. This idea was illustrated by two recent studies. Bachmann et al. (2019) showed that the selfer *C. orientalis* carries a nonfunctional *S*‐haplotype (*CoS_12_*) with defects in at least the *SCR* gene. While loss‐of‐function mutations are typically expected to be recessive (Kacser & Burns, 1981), *CoS_12_* actually confers a dominant SC phenotype when crossed with the closely related outcrosser *C. grandiflora*. Examination of the *CoS_12_* sequence revealed a functional orthologous haplotype in *C. grandiflora* that is relatively high in the dominance hierarchy of SI alleles. Closer inspection revealed that the *CoS_12_* haplotype contains a *mirS3* small RNA precursor whose mature small RNAs are predicted to silence the more recessive *C. grandiflora SCR* allele introduced by crossing. Hence, the fact that the nonfunctional *CoS_12_* is derived from a functional SI allele that was high in the dominance hierarchy may have caused the SC mutation to be dominant rather than recessive, and hence provided a selective advantage similar to Haldane's sieve (Haldane, 1927; Turner, 1981). It will now be important to derive theoretical models to formalize this idea and obtain precise predictions on the relative probability of SC mutations as a function of their level of dominance.

In a similar twist, the tetraploid *A. suecica* was formed by recent hybridization between the selfer *A. thaliana* and the outcrosser *A. arenosa.* Novikova et al. (2017) recently suggested that *A. suecica* became a selfer immediately upon hybridization because the nonfunctional SI allele it inherited from its *A. thaliana* parent (*S_A_* haplotype, again relatively high in the dominance hierarchy in Arabidopsis) is apparently still producing sRNAs predicted to be able to transcriptionally silence the more recessive *SCR* allele (*SCR*_2_) brought by its *A. arenosa* parent. Hence, the shift to selfing in this recently formed hybrid species may have been an indirect consequence of the identity (and dominance) of the SI alleles carried by the two progenitor parents. How much the evolution of the mating system of this hybrid species was contingent upon the dominance of the two particular SI alleles it inherited is an open question.

### 5.4 Open questions on molecular mechanisms of dominance

Small RNAs are involved in many different aspects of plant biology, ranging from developmental regulation and patterning to heterochromatin formation and the silencing of retroelements to disease resistance (Borges & Martienssen, 2015). A wide variety of different types of small RNAs that mediate these processes have been described; however, broadly speaking, they can be separated into micro‐RNAs (miRNAs) which are encoded by a hairpin precursor and target the mRNA of the target locus (post transcriptional silencing) and small interfering RNAs (siRNAs), which act in the RNA‐dependant DNA methylation (RdDM) pathway together with the specialized polymerases, PolIV and PolV to induce DNA methylation of the target loci and transcriptionally silence them (Matzke, Kanno, & Matzke, 2015). While the small RNAs involved in *S*‐locus pollen dominance encode miRNA‐like hairpin precursors, the sRNAs from the dominant *S*‐locus appear to function by targeting RdDM methylation to the promoter of the recessive *SCR* allele (at least in Brassica, Shiba et al., 2006). This is not without precedent, as previous reports have suggested that in some cases hairpin precursors can also target methylation using the RdDM pathway (Wu et al., 2010). In order to better understand how these dedicated sRNAs achieve silencing (Figure 4b), it will be important to first check if targeted DNA methylation of the recessive *S*‐alleles by the dominant *S*‐alleles is also found in Arabidopsis, and also to determine whether a canonical or a more unusual machinery of transcriptional silencing is involved. In addition, Durand et al. (2014) showed that the eight families of sRNA precursors regulating dominance among *A. halleri* SI alleles in pollen have arisen at different times in the course of evolution and thus evolved independently. They also have different predicted target sites and come in different sizes (21‐24nt). This raises the intriguing possibility that they may be using different mechanisms from those proposed in Brassica (and from each other). Finally, in contrast to the situation in pollen, *S*‐locus dominance in pistils does not appear to be due to differences in transcriptional regulation (of *SRK*) (Burghgraeve et al., 2018; Kusaba, Tung, Nasrallah, & Nasrallah, 2002; Suzuki et al., 1999) and may rather involve events occurring downstream such as protein dimerization (Naithani, Chookajorn, Ripoll, & Nasrallah, 2007). A forthcoming challenge in the field will be to resolve the molecular mechanism by which the dominance interactions have evolved in pistils.

BOX 1 **Louis Bernatchez: a personal encounter and enduring inspiration**

Perhaps the most enduring impact of Louis on my scientific endeavor is the recognition that science is a social activity. Arriving in his laboratory, as a young master student from France at the end of the winter of 1997 who knew close to nothing about Québec and so many things, one of the most vivid recollection I have is hearing Louis repeat with a Québec accent I was never able to master: “Tout’ se peut !” In other words, there are many ways to do science, and some can be far from your own fashion, but it is always OK to be yourself. This created around his person an enduring, living, and vibrant enthusiasm for research as a social activity that can bring people together.

Beyond personal encounters, his research program articulating ecological, evolutionary and genomic approaches around a model organism which he developed over the long time continues to be a rich source of inspiration. At this disciplinary interface, his open‐mindedness extended to the contemplation of scientific results: whatever counterintuitive the data might be, they are facts that must be considered in their full extent. He would also insist on the importance of investing the time and energy needed for methodological developments if they provide the answer to your question. Instead of limiting the data to just the “minimal publishable unit,” I saw him countless times encourage people to go straight for large datasets that went beyond the state of the art in their own field, even if they may seem too large at first glance. If large enough and well designed, they will allow serendipity and typically ended up raising unanticipated questions that in turn stimulated new methodological developments. In investing the time and energy, he would clearly preach by example.

The last lesson I would like to highlight here is the importance of always putting the students’ interest first. Every decision I saw him make was toward this goal. Being in Louis's laboratory was being part of an extended family, where you could be sure to be credited for your work. You would meet diverse people from around the world and be given the opportunity to develop your own ideas and projects. Your opinion would matter, and there would always be room for constructive discussion. So, when thinking about how to contribute to this invited review in his honor, I decided to keep the ball rolling and to invite my own current and former students, postdocs, some of which are now junior colleagues as well as some of my close collaborators to contribute. This recursive process is possibly the best homage I could pay to the unique impact Louis had on my scientific career.

Happy birthday Louis, et mille merci!

Vincent Castric

## 6 OUTSTANDING QUESTIONS ABOUT SELF‐INCOMPATIBILITY

In spite of intensive efforts to decipher the SI response, the molecular and physiological processes by which plants discriminate self‐ from nonself pollen remain incompletely described (Doucet, Lee, & Goring, 2016). Successful recapitulation of the SI reaction in the model *A. thaliana* by introduction of the *SCR* and *SRK* genes from the outcrossers *A. lyrata* (Nasrallah et al., 2002) provides a powerful platform to dissect the molecular mechanisms involved. A particularly puzzling observation was that *A. thaliana* orthologs of the candidate genes identified in Brassica for the downstream cascade can be inactivated in these SI *A. thaliana* lines with no major phenotypic consequences on the SI reaction (Kitashiba, Liu, Nishio, Nasrallah, & Nasrallah, 2011). Although this could be due to the heterologous nature of the *A. thaliana* model of SI, a more intriguing interpretation could be that these two species use distinct signaling pathways to fulfill the same apparent phenotype. Hence, a phenotypic stasis (the SI response) could be achieved in spite of extensive divergence of the underlying molecular components.

The field has so far mainly focused on a handful of model systems. There are good reasons for this, as the effort to clone and validate the role of SI genes remains a major challenge precisely because of their very function (inherently making the establishment of inbred lines impossible), and because of some of their most salient evolutionary properties (the high polymorphism and genomic complexity of the chromosomal regions where they lie), but it is clearly an objective worth pursuing for several reasons. Beyond the clear implications for applied research to facilitate the creation of stable varieties with agronomic characters of interest (Box 1), the comparison of a broader set of SI systems will enable the search for patterns of evolutionary convergence at the molecular level, both in terms of how plants discriminate self from nonself (Fujii et al., 2016) and how they transmit the signal at the cellular level and mount divergent physiological responses toward compatible and noncompatible pollen (Jany, Nelles, & Goring, 2019). The comparison of additional sporophytic systems should in particular determine whether the dominance interactions among *S*‐alleles can be controlled by molecular mechanisms that are different from the sRNA‐based mechanism identified in Brassicaceae (Durand et al., 2014; Tarutani et al., 2010). Hence, it is exciting that the ongoing efforts to clone SI genes out of the many SI species available in nature will enable a true overview of the diversity of molecular mechanisms that have been employed across the tree of life to fulfill that important biological function.

BOX 2 **Applied relevance of the field of research and your own work**

Although our own work is mostly geared toward fundamental research, understanding how SI is controlled and evolves has clear relevance for applied research. First, SI has been used as a way to control the production of F1 cultivars in Brassica crops (Nasrallah, 2004b). Second, the purpose of SI being the prevention of selfing inherently makes the production and maintenance of homozygous lines impossible. Hence, SI is also making the creation of new crop varieties more difficult for modern breeders, and constitutes a major hurdle that precision breeding is trying to by‐pass (Ye et al., 2018). Accordingly, selfing species may have been more prone to be domesticated than outcrossing species, and in return domestication may have favored the transition from outcrossing to selfing (Dempewolf, Hodgins, Rummell, Ellstrand, & Rieseberg, 2012). Third, SI can limit crop production, and the recent discovery of a SI system in olive trees with only two segregating SI alleles (Saumitou‐Laprade et al., 2010) calls for orchard management practices that take into account this potential limitation (Saumitou‐Laprade et al., 2017). Fourth, SI can contribute to put endangered species at increased risk of extinction, through the so‐called *S*‐allee effect (Wagenius et al., 2007), whereby the limit imposed by the SI system on seed production in small natural populations with low SI alleles diversity further contributes to demographic peril. Finally, we recently showed that population subdivision leads to greater invasion probability of SC alleles (Brom et al., 2020). In the near future, the ongoing fragmentation of most natural habitats is thus expected to lead to SI breakdown in many species, and thus evolution of the mating system.

Inbreeding depression is a key factor in to predict the evolution of mating systems in natural populations. At present, however, important aspects of its genetic architecture are crucially lacking. In particular, the distribution of fitness effects and dominance of segregating deleterious mutations as well as the contribution of the *S*‐linked genetic load relative to the overall genomic load, and how demographic variations and mating system shifts affect these factors in return typically remain poorly documented. Further theoretical and experimental studies will provide exciting novel insight to understand the factors favoring and maintaining SI or leading to SC and eventually selfing, and understand the evolutionary dynamics of *S*‐alleles.

## Data Availability

Data sharing is not applicable to this article as no new data were created or analyzed in this study.
